# *SCN1A* Channels a Wide Range of Epileptic Phenotypes: Report of Novel and Known Variants with Variable Presentations

**DOI:** 10.3390/ijms25115644

**Published:** 2024-05-22

**Authors:** Danai Veltra, Virginia Theodorou, Marina Katsalouli, Pelagia Vorgia, Georgios Niotakis, Triantafyllia Tsaprouni, Roser Pons, Konstantina Kosma, Afroditi Kampouraki, Irene Tsoutsou, Periklis Makrythanasis, Kyriaki Kekou, Joanne Traeger-Synodinos, Christalena Sofocleous

**Affiliations:** 1 Laboratory of Medical Genetics, Medical School, National and Kapodistrian University of Athens, St. Sophia’s Children’s Hospital, 11527 Athens, Greece; dveltra@med.uoa.gr (D.V.); kkosma50@gmail.com (K.K.); afrodite_kampouraki@yahoo.gr (A.K.); irenets78@hotmail.com (I.T.); pmakryth@med.uoa.gr (P.M.); kkekou@med.uoa.gr (K.K.); jtraeger@med.uoa.gr (J.T.-S.); 2 Research University Institute for the Study and Prevention of Genetic and Malignant Disease of Childhood, National and Kapodistrian University of Athens, St. Sophia’s Children’s Hospital, 11527 Athens, Greece; 3 Pediatric Neurology Department, St. Sophia’s Children’s Hospital, 11527 Athens, Greece; vitheodorou@yahoo.gr (V.T.); mkatsalouli@hotmail.com (M.K.); 4 Agrifood and Life Sciences Institute, Hellenic Mediterranean University, 71410 Heraklion, Greece; vorgiap@uoc.gr; 5 Pediatric Neurology Department, Venizelion Hospital, 71409 Heraklion, Greece; niotakisg@yahoo.gr; 6 Pediatric Neurology Department, Tzaneio Hospital, 18536 Piraeus, Greece; ftsaprouni@gmail.com; 7 First Department of Pediatrics, Medical School, National and Kapodistrian University of Athens, St. Sophia’s Children’s Hospital, 11527 Athens, Greece; roserpons@med.uoa.gr; 8 Department of Genetic Medicine and Development, Medical School, University of Geneva, 1211 Geneva, Switzerland; 9 Biomedical Research Foundation of the Academy of Athens, 11527 Athens, Greece

**Keywords:** epilepsy, genotype, phenotypic spectrum, sodium channelopathy, Dravet syndrome

## Abstract

*SCN1A*, the gene encoding for the Nav1.1 channel, exhibits dominant interneuron-specific expression, whereby variants disrupting the channel’s function affect the initiation and propagation of action potentials and neuronal excitability causing various types of epilepsy. Dravet syndrome (DS), the first described clinical presentation of SCN1A channelopathy, is characterized by severe myoclonic epilepsy in infancy (SMEI). Variants’ characteristics and other genetic or epigenetic factors lead to extreme clinical heterogeneity, ranging from non-epileptic conditions to developmental and epileptic encephalopathy (DEE). This current study reports on findings from 343 patients referred by physicians in hospitals and tertiary care centers in Greece between 2017 and 2023. Positive family history for specific neurologic disorders was disclosed in 89 cases and the one common clinical feature was the onset of seizures, at a mean age of 17 months (range from birth to 15 years old). Most patients were specifically referred for *SCN1A* investigation (Sanger Sequencing and MLPA) and only five for next generation sequencing. Twenty-six *SCN1A* variants were detected, including nine novel causative variants (c.4567A>Τ, c.5564C>A, c.2176+2T>C, c.3646G>C, c.4331C>A, c.1130_1131delGAinsAC, c.1574_1580delCTGAGGA, c.4620A>G and c.5462A>C), and are herein presented, along with subsequent genotype–phenotype associations. The identification of novel variants complements SCN1A databases extending our expertise on genetic counseling and patient and family management including gene-based personalized interventions.

## 1. Introduction

Epilepsy, a complex neurological disease characterized by spontaneous and recurrent seizures, affects 70 out of 100,000 individuals worldwide (~1–2% of global population). Classified into focal onset, generalized onset and unknown onset [1], the types of seizures can help define epilepsy as focal, generalized and combined focal and generalized, while when limited information hinders the determination of epilepsy as focal or generalized, it remains unknown. On a final level of diagnosis, epilepsy syndromes refer to clusters of features that tend to present together and may include specific seizure types, EEG and imaging findings and characteristic comorbidities such as neurodevelopmental deficits [2]. Treatment with antiepileptic medications (AEDs) is the first-line and the most common treatment of choice, completely controlling seizures in almost 70% of patients albeit not curing the underlying condition. In the remaining patients pharmaco-resistant epilepsy may be managed by surgery, electrical stimulation or dietary therapies (https://www.aans.org/en/Patients/Neurosurgical-Conditions-and-Treatments/Epilepsy, last assessed in 14 May 2024). The selection of proper approaches requires characterization and understanding of pathogenic mechanisms. As a complex disease, epilepsy extends to multiple levels of pathophysiology including inherent genetic alterations in the neurotransmission machinery components or acquired lesions linked to drug or other substance toxicity, strokes or infectious diseases and traumas/injuries [3]. Channelopathies, synaptopathies and interneuronopathies, as well as epigenetic dysregulation, neurodegeneration and mitochondrial deficiencies, are some of the genetic causes of epilepsy. Of those, channelopathies were for many years considered the prominent cause of epilepsy [4,5] and, although deposed in the presence of novel findings arising from next generation sequencing (NGS) approaches, still remain a major cause of variable types of epilepsy [6,7,8].

Voltage-gated Na^+^ channels share a common structure containing one α and one, or more, auxiliary β subunits. Amongst the nine well-characterized α subunit proteins, Na_v_1.1 is enriched in the central nervous system (CNS) and more specifically to the dendrites and cell bodies of excitatory neurons. Na_v_1.1 comprises four homologous domains (DI–DIV) each of which contains six α transmembrane helices (S1–S6) connected by loops. The S4 helix shapes the voltage sensors and the S5 and S6 helices, with their connecting loop, shape the ion-conducting pore [9]. *SCN1A*, the gene encoding for the Nav1.1 channel, has 26 exons, is located at 2q24.3 and exhibits dominant interneuron-specific expression. *SCN1A* pathogenic or likely pathogenic variants affect the initiation and propagation of action potentials and disrupt regular neuronal excitability, leading to various types of seizures [10]. Depending on the actual impact of each variant they can be specifically categorized as loss of function (LOF), partial loss of function (pLOF), decreased excitability (DE), gain of function (GOF), increased excitability (IE) and simultaneously gain–loss of function (G-LOF). Genotype–phenotype correlations have indicated that LOF variants affecting the pore region are mainly associated with SMEI although missense variants in the same region have been recorded in patients with generalized epilepsy febrile seizure + (GEFS+) [11,12]. Focal febrile seizures are usually linked to pLOF and G-LOF variants and GEFS+ to IE, DE, pLOF or GOF variants [12]. *SCN1A* variants are transmitted in an autosomal dominant pattern; however, incomplete penetrance and variable expressivity have been recorded, further adding to the great variability of phenotypes, the first of which is currently known as Dravet syndrome, previously SMEI, as described by the French neurologist Charlotte Dravet in 1978 [13]. Advances in the clinical description and genetic diagnosis of many patients has allowed the classification of a broad spectrum of *SCN1A*-related disorders, including DS, myoclonic-atonic epilepsy (MAE), epilepsy of infancy with migrating focal seizures (EIMFS), early-infantile developmental and epileptic encephalopathy (EIDEE), GEFS+ and partial epilepsy with febrile seizure plus (PEFS+), as well as non-epileptic disorders such as familial hemiplegic migraine (FHM), autism spectrum disorders (ASD) and arthrogryposis [14,15,16,17,18]. SCN1A-related disorders are somehow considered pharmaco-resistant, and the use of sodium channel blockers, such as phenytoin, carbamazepine, oxycarbazepine and lamotrigine, is contraindicated in patients with channel haploinsufficiency, where aggravation of seizures and consequent neurodevelopmental deficits have been observed [19]. Treatment with topiramate or valproic acid [20] or enhancement of GABA receptor activity via benzodiazepines or stiripentol present a better option which combined with a ketogenic diet seems to have better results particularly in respect to cognition [21].

To date, more than 2000 *SCN1A* variants have been recorded (Human Gene Mutation Database Gen-Locus Specific Database https://www.hgmd.cf.ac.uk/ac/index.php last assessed in 14 May 2024, and SCN1A database http://scn1a.caae.org.cn/index.php, last assessed in 14 May 2024) and associated with variable phenotypes. Besides a rather clear correlation between truncating loss of function variants and more severe presentations, further genotype–phenotype associations remain open to question [11,20].

Findings, and subsequent genotype–phenotype associations, from a cohort of patients referred for *SCN1A* genetic testing are herein presented to enrich *SCN1A* databases, exchange acquired knowledge and enhance our expertise on patient diagnosis, prognosis and management. This is particularly significant for differential diagnosis in the extremely clinically heterogeneous SCN1A-related disorders.

## 2. Results

**Figure 1:**
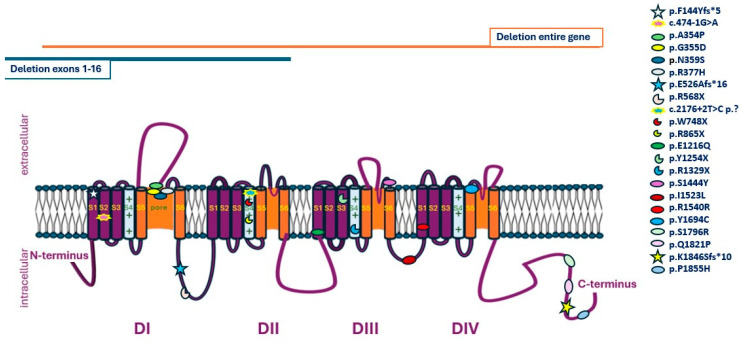
Schematic representation of *SCN1A* variants disclosed in the current cohort.

Twenty-five variants were detected, nineteen with Sanger sequencing, one with multiplex ligation-dependent probe amplification (MLPA), one after clinical exome sequencing (CES) and four after whole exome sequencing (WES). Of those, nine were novel, with no previous reports in the literature or databases available (Families 1, 3, 5, 10, 14, 15, 19, 22 and Table 1). Eleven variants were missense, six nonsense, three frameshift, two splice site, one synonymous, one partial gene deletion and one whole gene deletion (Figure 1). Subsequent segregation analysis and family studies were available in 19 families and disclosed the de novo nature in 12 cases.

Twenty-five patients (12 males, 13 females; mean age at referral: 4.5 years; 6 months–31 years), from 24 unrelated families, received a diagnosis of an SCN1A-related channelopathy. The earliest seizure onset was recorded in P22 on the fifth day of life and the latest in P5 and P13 at the age of 2 years (median age of onset 7.8 months). Well-recognized triggers of seizures included fever, infections and vaccinations while first symptoms varied from seizures (10), febrile seizures (5), febrile status epilepticus/complex seizures (5) and combinations of febrile and afebrile seizures (5).

**Figure 2:**
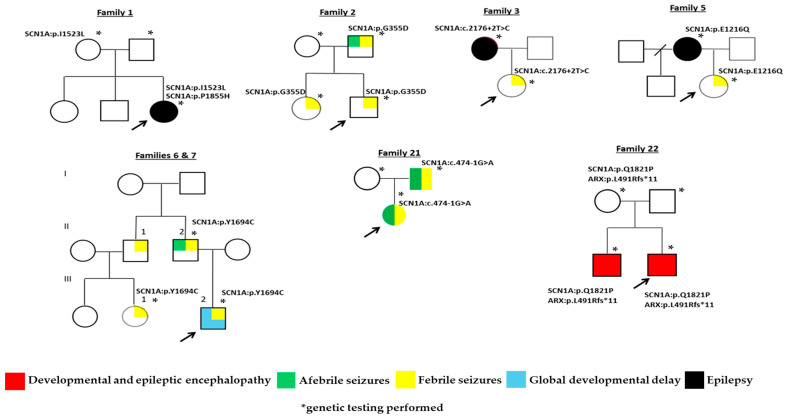
Family trees of cases with transmission of pathogenic or likely pathogenic *SCN1A* variants.

Uneventful pregnancies and perinatal periods were reported for all patients while a family history of seizures or epilepsy was positive for 15 cases. The father of Patient 2 had febrile and afebrile seizures and transmitted the SCN1A:c.1064G>A variant to both his offspring, who had only febrile seizures. In Family 3, the patient experiencing her first episode of febrile seizures (FS) at the age of 6 months, while having a COVID-19 infection, inherited the SCN1A:c.2176+2T>C variant from her mother who experienced seizures between ages 6 and 15 years. The mother of Patient 5 reported epilepsy between ages 6 and 7 and has transmitted the SCN1A:c.3646G>C variant to her daughter with the onset of complex FS at age 2 during an upper respiratory infection. In Families 6 and 7, first cousins have inherited the SCN1A:c.5081A>G variant form their fathers who are brothers and both report seizures. Patient 21, a 10-month old female with status epilepticus (25 min long) during COVID-19 infection shared the same SCN1A:c.474-1G>A variant with her father (Figure 2). Table 2 summarizes data on the clinical presentation and the variants detected in all 25 cases.

In respect to neurodevelopment, some patients presented with disorders affecting intellectual abilities including speech and/or motor skills and disorders within the autism spectrum (Patients 3, 6, 9–11, 15, 17–18, 20, 22–24).

## 3. Discussion

Consistent with the core phenotypic features ofDS, the first clinical presentation linked to *SCN1A* [13], 20/25 probands experienced their first seizure during infanthood and 16/25 had febrile seizures. In three patients, a genetic diagnosis was achieved immediately after the first episode and before the development of the complete clinical presentation. In older patients, additional types of seizures were recorded along with cognitive and learning difficulties. Triggers of a first episode included infections such as COVID-19, influenza Β or infections of the upper respiratory system (five patients), as well as vaccinations.

Cases of special interest comprise co-segregation of two variants either in *SCN1A* {p.(P1855H)/p.(I1523L) in Family 1} or *SCN1A* and *ARX* genes (Family 22). In the first case, p.(P1855H) is a pathogenic de novo variant considered causative, and p.(I1523L) maternally inherited and likely pathogenic. Recessive inheritance of *SCN1A* variants has been described before, in cases with high levels of consanguinity and variable clinical presentations, similar to those recorded in autosomal dominant transmission [23,24,25]. Variants detected were considered hypomorphic, interfering with *SCN1A* function and decreasing seizure threshold when combined. Studies of transgenic mouse models for the p.(R1648H) variant showed that the negative effect of the variant is somehow amplified in cases of homozygosity where mice suffer spontaneous generalized seizures and have a reduced life span [25]. In Patient 1, identification of the variants’ orientation as trans or cis was unsuccessful, hence hindering further evaluation of the possible clinical implications of a compound heterozygosity. Proline at position 1855 is very highly conserved and considered important for the protein function [26] and, although it is a novel variant, another substitution from proline to leucine p.(P1855L) has been previously recorded and reported as associated with DS [27]. SCN1A:p.I1523L is also a novel variant however a different substitution affecting the same amino acid, p.(I523T), has been recorded in a female patient with FS onset at 6 years of age, inducing developmental delay, and evolving to tonic–clonic and SE in adulthood [28].

In case 22, the recorded DEE was attributed to an *ARX* (MIM#300382) pathogenic frameshift variant in the context of the well described DEE type 1 (MIM#308350) [29]. Despite the evident association of the *ARX* variant with the severe phenotype of both siblings, the impact of SCN1A:p.Q1821P, a likely pathogenic maternal variant, remains elusive and may require further analysis. According to large patient series, some 3.2–7.2% may have multiple molecular diagnoses where different variants segregating independently are associated with distinct clinical entities with specific or overlapping presentations. Complex interactions between the different effects of pathogenic variants detected in more than one gene in a certain individual result in “blended phenotypes”, a term conceived to represent mixed clinical presentations [30]. Variants detected in epilepsy-related genes (*DEPDC5, CHD2, SCN8A* and *IQSEC2*) have been reported in patients with *SCN1A* variants and were considered to contribute to blended phenotypes comprising features of the disorder associated with the epilepsy gene and Dravet syndrome [31]. In general, the presence of possibly causative variants in phenotypically unaffected parents perplexes final classification and diagnosis. For SCN1A-related disorders, 10% of parents with (likely) pathogenic variants remain asymptomatic while offspring inheriting variants from affected parents have less than a 100% chance of developing seizures both due to incomplete penetrance and variable expressivity (https://www.ncbi.nlm.nih.gov/books/NBK1318/, SCN1A seizure disorders, Gene Reviews, last assessed in 14 May 2024). As with cases 1 and 22 the presence of variants in the apparently healthy mothers may complicate case resolution but should not be overlooked, especially in respect to prognosis, calculated risks for future pregnancies and genetic counseling.

Genotype–phenotype correlation studies assess variants in the context of type, location and impact on the channel produced including functional distribution of missense variants and truncation effects on disease onset [27]. Those affecting the crucial voltage sensor S4, or pore S5–S6 domains or their intermediate loop typically lead to severe phenotypes with multiple types of pharmaco-resistant seizures and neurodevelopmental delay [12]. In Families 11, 12 and 23, probands presenting with global developmental delay, albeit of variable severity possibly explained by their different developmental stages, all have nonsense variants affecting the S4 subunit. Variants affecting subunits S5–S6 and their connection loop were detected in Families 2, 6/7, 9, 10, 14 and 25 where, except for cases 9 and 25, a positive history for various types of seizures was reported. However, segregation analysis, available in all but Family 14, indicated transmission of variants only in Families 2 and 6/7 (Figure 2). Phenotypic variability was noted in Families 6/7, where confounding mild global developmental delay and febrile status epilepticus were the reasons for referral in the proband (III-2, Figure 2). The offspring of a male patient had a diagnosis of grand mal epilepsy and seizures initially febrile and then afebrile until the age of 18 years old. His brother (II-1, Figure 2) also reports persistent but non frequent (1–2 episodes annually) febrile seizures treated with sodium valproate and, although refusing genetic testing, seems to have transmitted the familial pathogenic variant to his daughter (III-1, Figure 2), diagnosed immediately after her first episode due to the positive family history.

Variants leading to haploinsufficiency, owing to the partial or complete loss of *SCN1A,* comprised deletions previously described and associated with increased severity. The 157.5kb deletion encompassing the *SCN1A* gene and a deletion including exons 1–16 were detected in Families 16 and 24, respectively. DEE, which is the most severe end of the *SCN1A*-related disease spectrum, was evident in both families, while in Family 24 additional clinical features, such as polydactyly and syndactyly (present also in the mother), were attributed to a *GLI3* heterozygous pathogenic variant of maternal origin. Further supportive of the deleterious effect of abolishing gene function, frameshift or nonsense LOF variants affecting even regions outside S4, S5 and S6 helices were also detected in Families 4, 8, 15, 17, 18 and 20 where neurodevelopmental disorders such as GDD, DEE and ADHD were prominent (Table 2).

Splice site variants affecting nucleotides on ±1 or ±2 positions are widely annotated as LOF and usually considered diagnostic [32]. The SCN1A:c.474-1G>A variant, detected in Family 21, is an already known splice site variant, previously recorded de novo in a patient with classic DS [27]. Affected members of Family 21 (father and proband) present with what may seem to be clinically heterogeneous, whereby the father reports the onset of multiple seizures after a head injury at age 1 year old and through childhood while his daughter experienced the onset of afebrile focal and febrile generalized tonic–clonic seizures at 4.5 months and a status epilepticus during COVID-19 infection. WES disclosed no additional variants in epilepsy related genes. The SCN1A:c.2176+2T>C novel variant detected in Family 3 is also inherited and associated with clinical heterogeneity. The mother reports normal neurodevelopment and seizures between childhood and adolescence. Her daughter, now four years old, has motor and speech difficulties and experienced the first episode of febrile seizures at the age of 6 months during COVID-19 infection. Further supportive of variable expressivity, SCN1A:c.2176+2T>A affecting the exact same nucleotide was recorded in a patient with onset of afebrile seizure at age 5.1 months after Diphtheria-Tetanus-Pertussis (DTP) vaccination [33]. Of interest, despite the positive family history, in both cases, a genetic diagnosis was sought and achieved only after hospitalization, indicating that if not admitted they might have ended up undiagnosed.

Missense variants are always difficult to interpret, especially if no previous records and functional studies are available. In silico assessments may support final classification while segregation analysis may prove valuable when the variant is characterized either de novo in the proband or transmitted from an affected parent. The SCN1A:p.S1796R variant detected in Patient 13 affects a very highly conserved neutral serine on position 1796 at the C-terminus of the channel. Positively charged hydrophilic arginine presents an unfavored substitution and combined with its de novo presence seems to explain the complex febrile and afebrile seizures. SCN1A:p.E1216Q detected in proband 5 and her mother was deemed relevant to the clinical presentation of febrile and afebrile seizures. The variant is novel and according to the *SCN1A* database only one variant, a truncating p.(E1216X), on the same position has been recorded providing limited details on the possible effect of corresponding missense variants [34]. In respect to amino acid propensities the highly conserved glutamic acid, on position 1216, is hydrophilic with a negative charged side chain and important for the channel’s function [26]. The glutamine variant has a neutral side chain and may require functional studies towards better understanding of underlying mechanisms. Patient 10 presents with seizures and attention deficit hyperactivity disorder (ADHD) and has the de novo novel p.(S1444Y) variant. Functional data on the S1444Y variant are lacking and limited to in silico assessments. Serine 1444 is a conserved (although not highly) neutral residue, the substitution of which by tyrosine with an aromatic side chain is considered deleterious and associated with the disease [26]. The family reports seizures from the matrilineal lineage (maternal sister and niece). Targeted molecular analysis for the patient’s parents failed to disclose the variant and no further segregation was recommended. Families with a positive history of seizures and pathogenic variants detected de novo only in one proband are recorded in other cohorts as well [11]. Among genetic or non-genetic factors that could explain the unexpected absence of the specific variant in all affected family members, the well-documented phenomenon of parental mosaicism (reaching 8.6% in *SCN1A* [35]), as well as the possibility of diagnostic pitfalls of Sanger sequencing including human and technical errors, should be taken into account [36,37].

In Family 14, where two first cousins report febrile seizures, the parents refused to be tested, hence the de novo or inherited nature of the R377H variant could not be evaluated. Arginine 377 is a highly conserved hydrophilic variant for which a previously reported substitution by the neutral glutamine has been associated with GEFS+ or epilepsy and/or neurodevelopmental disorder (NDD) [38,39,40,41]. Histidine is also hydrophilic and might represent a better tolerated variant associated with milder presentations. The SCN1A p.R1540R synonymous variant was one of the first variants detected in this study. Characterized as appearing de novo in proband 19, it could only be assessed in silico, where Human Splicing Finder 3 [42] indicated that it possibly affects splicing through the breaking of the exonic splicing enhancer and silencer (ESEs and ESSs). Previous reports of synonymous variants with a pathogenic impact include the SCN1A:c.693A>T:p.P231P variant detected in a patient with generalized myoclonic seizures. P231P was considered pathogenic due to a predicted negative effect on normal splicing [43]. R1540R is currently re-classified as a variant of unknown significance for which further studies are needed to establish a robust association with the observed phenotype.

Clinical heterogeneity is a well acknowledged characteristic of SCN1A-related disorders whereby different presentations may be recorded even in family members with the same variant [11,44,45]. Concepts like incomplete penetrance and variable expressivity are most likely based in biology where genetic, including digenic, oligogenic and polygenic inheritance as well as double-hit contributions, and epigenetic factors are important players [30]. The impact of truncating variants on early seizure onset, and of the distribution of missense variants (depending on the functional importance of each area) may somehow explain the different clinical expression of variants. However, the phenotypic variation in identical genotypes, shown even by variable responses to antiepileptic treatment such as valproic acid, remains elusive [45]. Drug resistance in several cases indicates endogenous and somehow personalized responses possibly affected by modifying genetic and epigenetic factors. Genetic modifiers, distinct genes regulating gene expression and disease severity, include *HLF*, *GABRA2*, *SCN2A*, *KCNQ2* and *SCN8A* and were identified as influencing phenotypic expression in mice models [46].

Investigation of epileptic phenotypes with NGS is expected to identify such additional genetic modifiers in cases with an *SCN1A* variant as well as detect a causative variant in negative cases. Whole exome sequencing was performed in 10 patients, where targeted *SCN1A* analysis disclosed no pathogenic variants. Pathogenic or likely pathogenic variants explaining the observed phenotypes were disclosed in five, comprising one in *TGM6*, one in *NR2F1*, one in *AFF3* and two in *PCDH19* [22], a gene known as a Dravet mimicker [15]. Dravet mimic genes, reported to cause phenotypes resembling DS, recorded up today include *SCN2A, SCN8A, SCN9A, SCN1B, PCDH19, GABRA1, GABRG2, STXBP1, HCN1, CHD2 *and *KCNA2*. These may also associate with distinct clinical presentations likely to shape novel clinical entities [15].

For many years now, channelopathies, mainly represented by *SCN1A* channelopathy, have been considered the prominent cause of epilepsy, a theory that seems to fade with advanced understanding of epileptogenesis. *SCN1A*-related disorders do cover a wide spectrum of clinical presentations, ranging from familial hemiplegic migraines to severe developmental epileptic encephalopathy, highlighting the variable expressivity and incomplete penetrance recorded with this gene. Targeted *SCN1A* Sanger sequencing, compensated by the Greek National Health System for hospitalized patients, has led to over-referrals and low diagnostic rates. Widely available NGS techniques, facilitating robust diagnosis and the additional identification of novel genes should become the first tier approach, proving to even become more cost-effective for national health systems.

Ongoing research in the field of epilepsy focuses on the identification of variants and predicted disease outcomes through in silico and functional assessments. Previous studies [47] have shown that, regardless of the reported phenotype, conventional in silico software correctly characterized benign from pathogenic variants in nearly 90% of cases, even if not able to differentiate disease severity (DS vs. GEFS+ vs. FHM), whereas patch-clamp mammalian expression systems analysis allowed evaluation of functional differences and genotype–phenotype correlations among missense variants. Braunkalus et al. [48] developed a prediction model to allow objective prognosis of disease course to DS or GEFS+ and although with expected difficulties concerning genetic and epigenetic modifiers this is expected to further assist disease management. Detection and characterization of variants is of paramount importance especially in view of emerging therapeutic approaches. Antisense oligonucleotides restoring functional SCN1A mRNA, adeno-associated virus delivered gene regulation and dCas9-based SCN1A gene activation are some of the genomics-driven therapies focusing on seizure control in SCN1A-related therapies [49,50,51].

In this context, and despite limitations concerning the small number of variants and the unavailability of extensive follow-up and functional assessments, findings from this current study are reported to enrich the *SCN1A* variant database and highlight related phenotypes. Acknowledgement of variants detected in patients with an SCN1A-related disorder facilitates diagnosis, prognosis and genetic counseling especially with respect to recurrence and disease risks [11,52]. Advanced understanding of *SCN1A* genotype–phenotype associations promotes development of gene-based interventions and better disease management crucial for the patients and their families.

## 4. Materials and Methods

### 4.1. Patients

This study was approved by the institutional boards and Ethics Committees of the National & Kapodistrian University of Athens and the St. Sophia’s Children’s Hospital (approval number is 12160/23-05-16). Written informed consent was obtained from all participants or their legal guardians. The study’s cohort included 343 patients (167 female and 176 male) referred by physicians in hospitals and tertiary care centers from the Hellenic territory, between 2017 and 2023. Positive family history for specific neurologic disorders was disclosed in 89 cases and is summarized in Table 3. The one common feature of all patients was the onset of seizures, at a mean age of 17 months (range from birth to 15 years old).

**Figure 3:**
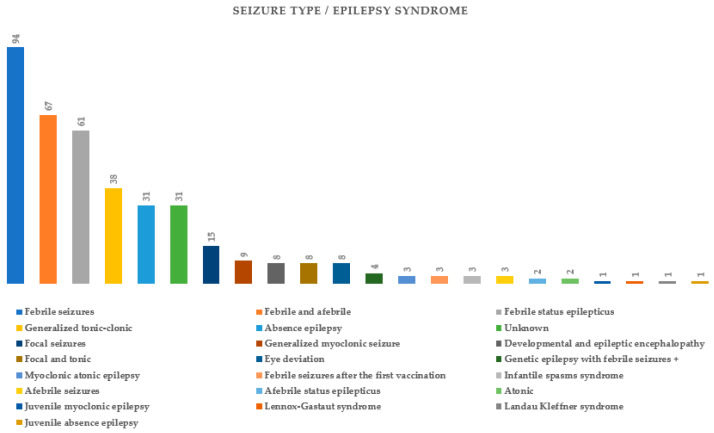
Seizure types or diagnosed epileptic syndromes in probands tested for *SCN1A* variants.

**Figure 4:**
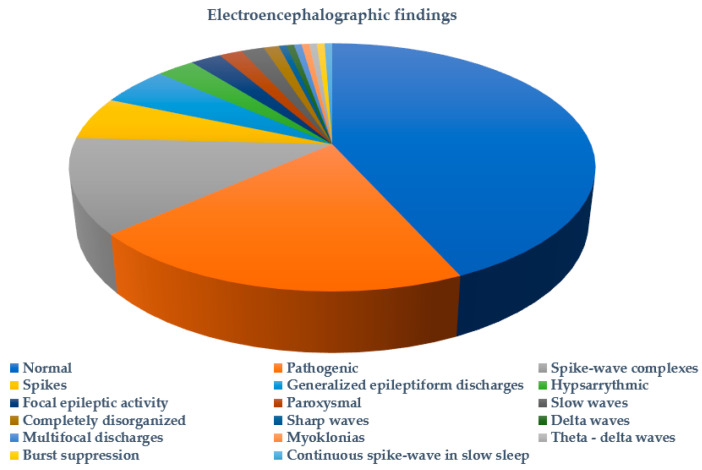
Electroencephalographic findings in probands tested for *SCN1A* variants.

**Figure 5:**
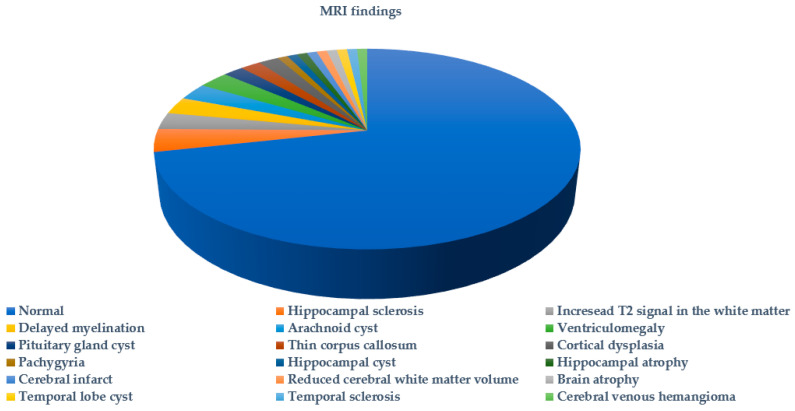
MRI findings in probands tested for *SCN1A* variants.

Figure 3 summarizes the types of seizures or epileptic syndromes recorded. Electroencephalographic findings were available for 51.89% (178/343) of probands and imaging for 32.06% (110/343) (Figure 4 and Figure 5 respectively). Other clinical findings included: global developmental delay (82/343, 23.90%), speech delay (22/343, 6.41%), ASD (21/343, 6.12%), microcephaly (8/343, 2.33%), disorders of intellectual development (7/343, 2.04%), hypotonia (7/343, 2.04%), coarse facial features (6/343, 1.74%) and macrocephaly (2/343, 0.74%). Poor fine motor coordination, plagiocephaly, trigonocephaly, hydrocephalus, macroglossia, hypertonia, tremor, hemiplegia, ataxia, exostoses, cryptorchidism, hypogonadism, hyponatremia and enuresis nocturna were also reported in isolated cases.

### 4.2. Methods

Genomic DNA was extracted from peripheral blood lymphocytes with the QIAsymphony^®^ DSP DNA technology (QIAGEN^®^, Hilden, Germany) according to the manufacturer’s protocols. Sanger sequencing for *SCN1A* was applied in 339 samples using the BigDye^TM^ Terminator sequencing kit (Thermo Fisher Scientific, Waltham, MA, USA) and ABI Prism 3500 Genetic Analyzer (Applied Biosystems, Woburn, MA, USA). For CNV detection, Multiplex ligation dependent probe amplification (MLPA^®^, kit P137, by MRC Holland, Amsterdam, The Netherlands) was performed in 41 samples (22 female and 19 male).

Whole exome sequencing (WES), performed for 4 probands, used the Human Core Exome (Twist Bioscience^®^, San Francisco, CA, USA) or xGenTM ExomeResearch v2 (Integrated DNA Technologies, Coralville, IA, USA) and CES was performed for 1 proband with the Clinical Exome Solution kit (by SOPHiA Genetics SA, Rolle, Switzerland) following the manufacturer’s recommendations. The resulting libraries were subjected to paired-end sequencing on an Illumina NextSeq^®^ 500 (Illumina, San Diego, CA, USA). Library preparation and sequencing were outsourced (Genotypos-Science Labs, Athens, Greece, and NIPD Genetics, Nicosia, Cyprus). ES data quality acceptance parameters included a mean depth of coverage >50×, with >98% regions at 25×. Variant annotation was performed with the ANNOVAR algorithm and variant filtration with the VarAFT v2.16 Variant Analysis and Filtration Tool (http://varaft.eu, last assessed in 14 May 2024), or the VarSome Clinical platform (https://clinical.varsome.com/ last accessed on 14 May 2024) (Saphetor SA, Lausanne, Switzerland). Variant filtration was made for *SCN1A* gene (GRCh37/hg19) and CNV detection used the ExomeDepth software version 1.1.15 [53].

Variant classification followed the recommendations of the American College of Medical Genetics (ACMG) [32] and CNV classification followed those of the ACMG and Clinical Genome Resource (ClinGen) [54]. When appropriate, confirmation of biparental inheritance of short tandem repeat (STR) markers allowed the use of PS2 criterion for de novo variants.

## Figures and Tables

**Table 1:** Characteristics of the novel variants detected in the current cohort.

Variant (Reference Transcript GRCh37: NM_001165963.4)	Population Frequency	GnomAD	VarMap	SIFT	Revel	Location in the Channel	Expected Impact on Protein Function
c.4567A>Τ:p.I1523L	-	-	Likely deleterious (0.39)	0.001	0.777	DIII–DIV loop	Unknown
c.5564C>A:p.P1855H	-	-	Likely deleterious (0.94)	0	0.939	C-terminal	Unknown
c.2176+2T>C	-	-	N.A.	N.A.	N.A.	DII, S4	LOF
c.3646G>C:p.E1216Q	-	-	Likely deleterious (0.59)	0	0.909	DIII, S1	Unknown
c.4331C>A:p.S1444Y	-	-	Likely deleterious (1.02)	0	0.894	DIII, loop S5–S6	Unknown
c.1130_1131delGAinsAC:p.R377H	-	-	N.A.	N.A.	N.A.	DI, loop S5–S6	Unknown
c.1574_1580delCTGAGGA:p.E526Afs*16	-	-	N.A.	N.A.	N.A.	Loop DI–DII	LOF
c.4620A>G:p.R1540R	-	-	N.A.	N.A.	N.A.	DIV, S1	Unknown
c.5462A>C:p.Q1821P	-	-	Likely deleterious (0.39)	0.001	0.893	C-terminal	Unknown

Abbreviations: N.A.: non-applicable.

**Table 2:** Data on the clinical presentation and variants detected in cohort’s probands.

P	G	Age of Diagnosis	Variant (Reference Transcript GRCh37: NM_001165963.4)	ACMG/ ACMG and ClinGen Classification (for CNVs)	Inheritance	Clinical Presentation	EEG	MRI	Treatment
1	F	1.5 y	c.4567A>Τ p.I1523L	Likely pathogenic (PM1, PM2, PP2, PP3)	Maternal	Seizure onset at 1 y.	Normal.	Increased Τ2 and FLAIR signals in the subcortical white matter in the frontal lobes.	LEV, VPA.
			c.5564C>A p.P1855H	Pathogenic (PS2, PM1, PM2, PM5, PP2, PP3)	De novo				
2	M	7 m	c.1064G>A p.G355D	Pathogenic (PM1, ΡΜ2, ΡΡ2, PP3, PP5)	Paternal	FS at 7 m.	N.A. Diagnosis after first seizure.	N.A.	N.A.
3	F	11 m	c.2176+2T>C	Pathogenic (PVS1, PP3, PM2)	Maternal	FS at 6 m during COVID-19 infection Gait with support. Speech delay.	Normal.	N.A.	N.A.
4	M	8 y	c.5536_5539delAAAC p.K1846Sfs*10	Pathogenic (PVS1, PP3, PP5, PM2)	N.A.	FSE at 4.5 m. Currently generalized tonic–clonic FS and AFS. SASD. Multiple exostoses syndrome.	Normal.	Normal.	LEV, VPA, RIS.
5	F	3 y	c.3646G>C p.E1216Q	Pathogenic (PΜ1, PM2, PP2, PP3, PM5)	Maternal	Complex FS during an upper respiratory infection. Seizure onset 2 y.	N.A.	Normal.	VPA.
6	M	1.5 y	c.5081A>G p.Y1694C	Likely pathogenic (PM1, PM2, PP2, PP3, PP5)	Paternal	FSE at 1.5 y. Mild GDD. Speech and occupational therapy.	N.A. Diagnosis after first seizure.	N.A.	VPA.
7	F	9 m	c.5081A>G p.Y1694C	Likely pathogenic (PM1, PM2, PP2, PP3, PP5)	Paternal	FS onset at 9 m.	N.A. Diagnosis after first seizure.	N.A.	N.A.
8	F	1 y	c.429_430delGT p.F144Yfs*5	Pathogenic (PVS1, PM1, PP3, PP5)	N.A.	Multiple episodes of FSE. Seizure onset at 8 m.	Normal.	Normal.	LEV, VPA.
9	M	6 y	c.1060G>C p.A354P	Pathogenic (PS2, PΜ1, PM2, PP2, PP3)	De novo	DEE onset at 6 m.	Pathogenic.	N.A.	LEV, VPA, CLB.
10	M	3 y	c.4331C>A p.S1444Y	Pathogenic (PS2, PM2, PP2, PP3, PP5)	De novo	FS onset at 14 m. One SE 1 h long. Attention deficit disorder.	Normal.	Normal.	N.A.
11	M	4 y	c.4933C>T p.R1645X	Pathogenic (PVS1, PM2, PP3, PP5)	N.A.	FS and AFS: onset at 7 m. Currently monthly attacks of focal seizures (3 min long). Mild GDD. Speech and occupational therapy.	N.A.	Normal.	VPA.
12	F	3.5 y	c.2593C>T p.R865X	Pathogenic (PS2, PVS1, PP3, PP5, PM2)	De novo	Seizures after vaccination (DTP). Multiple daily episodes of absence seizures and myoclonus.	Myoclonus and photosensitivity.	Normal.	PB, VPA, LTG, CLB.
13	F	3 y	c.5388T>A p.S1796R	Pathogenic (PS2, PM1, PM2, PP2, PP3, PP5)	De novo	Complex FS and AFS onset: 2 y.	N.A.	N.A.	N.A.
14	F	2 y	c.1130_1131delGAinsAC p.R377H	Pathogenic (PM1, PM2, PM5, PP2, PP3)	N.A.	Multiple FS episodes. Diagnosis during Influenza B infection.	N.A.	N.A.	N.A.
15	F	18 y	c.1574_1580delCTGAGGA p.E526Afs*16	Pathogenic (PS2, PVS1, PM2, PP3, PP5)	De novo	Generalized tonic–clonic seizures: onset at 7 m. ADHD. Clumsiness. High IQ.	Initially pathogenic and then with photosensitivity. Now normal.	Venous hemangioma of the pituitary gland.	Now without treatment. Previously seizure aggravation was reported with the use of CBZ.
16	F	5 y	chr2:166,848,222–167,005,693del size: 157.5 kb contains the whole *SCN1A* gene.	Pathogenic (1) (2B, 2C, 4L)	N.A.	FS and AFS, focal to secondary generalization, episodes of SE, myoclonic atonic seizures. Onset at 6 m. Hemiparesis.	Normal.	Normal.	LEV, VPA. Seizure aggravation was reported with the use of OXC.
17	M	2.5 y	c.3762T>A p.Y1254X	Pathogenic (PS2, PVS1, PM2, PP3, PP5)	De novo	Multiple episodes of FS and AFS: onset at 5 m. 3 SE. Action tremor.	Nonspecific findings.	Normal.	LEV, VPA, TPM.
18	M	2 y	c.1702C>T p.R568X	Pathogenic (PS2, PVS1, PM2, PP3, PP5)	De novo	Multiple episodes FS and AFS: onset at 3.5 m. Multiple FSE. Eyelid myoclonia. GDD.	Pathogenic.	Atrophy of the left temporal lobe with progressive atrophy and gliosis/sclerosis of the associated hippocampus.	LEV, VPA, LCM.
19	F	2 y	c.4620A>G p.R1540R	VUS (PS2, PM2)	De novo	FS: onset at 8 m. SE: onset at 1 y. Absence seizures.	Delta waves.	Normal.	LEV.
20 ^‡^	M	31 y	c.2244G>A p.W748X	Pathogenic (PVS1, PM2, PP5)	De novo	DEE: onset at 7 m. Pharmaco-resistant epilepsy. IQ < 30. Gait difficulties. 4–5 words vocabulary. Patient deceased after a respiratory and urogenital infection.	N.A.	N.A.	N.A.
21 ^‡^	F	10 m	c.474-1G>A	Pathogenic (PVS1, PP5, PM2)	Paternal	Afebrile focal and febrile generalized tonic–clonic seizures: onset 4.5 m. SE (25 min) during COVID-19 infection.	N.A.	N.A.	LEV, VPA, CLB.
22 ^‡,^^	M	6 m	c.5462A>C p.Q1821P	Likely pathogenic (PM2, PP2, PP3)	Maternal	DEE (seizure onset at 5 days). Hypotonia.	Pathogenic.	Hypoplasia of corpus callosum.	PB, LEV.
			*ARX* gene NM_139058.3: c.1472delT: p.L491Rfs*11	Pathogenic (PVS1, PM2, PP3)					
23 ^‡,^^	M	13 y	c.3985C>Τ p.R1329X	Pathogenic (PVS1, PM2, PP3, PP5)	N.A.	DEE (seizure onset 5.5 m).	N.A.	Normal.	LEV, VPA.
24 ^‡‡,^^	M	1.5 y	Chr2:166,892,579–167,005,693del (includes exons 1–16 of *SCN1A* gene)	Likely pathogenic (0.9–1A, 2B, 2C, 3A)	De novo	DEE (seizure onset at 2 m). Hexadactyly. Syndactyly.	Normal.	Normal.	LEV, PHT.
			*GLI3* gene NM_000168.6:c.793delG:p.A265Pfs*45	Pathogenic (PVS1, PM2, PP3)					
25	F	1 y	c.1076A>G p.N359S	Pathogenic (PS2, PM1, PM2, PM5, PP3, PP5)	De novo	FSE (4 times, onset 8 m) first episode during COVID-19 infection, second after Streptococcus pneumoniae vaccination and the fourth after RSV infection.	Normal.	N.A.	LEV, VPA.

Footnotes ^‡^ Diagnosed after WES; ^‡‡^ Diagnosed after CES; ^^^ included in a previous report [22]. Abbreviations P: proband number; G: gender; M: male; F: female; y: years; m: months; FS: febrile seizures; AFS: afebrile seizures; FSE: febrile status epilepticus; DTP: diphtheria–tetanus–acellular pertussis adsorbed–inactivated poliomyelitis; DEE: developmental and epileptic encephalopathy; ASD: autistic spectrum disorder; CBZ: carbamazepine; CLB: clobazam; LCM: lacosamide; LEV: levetiracetam; LTG: lamotrigine; OXC: oxcarbazepine; PB: phenobarbital; PHT: phenytoin; RIS: risperidone; TPM: topiramate; VPA: valproate acid; N.A.: non-applicable.

**Table 3:** Positive family history recorded in the cohort.

Febrile seizures	19.53% (67/343)
Epilepsy	17.20% (59/343)
ASD	0.87% (3/343)
Intellectual development disorder	0.87% (3/343)
Psychiatric disorders	0.58% (2/343)
Encephalitis	0.29% (1/343)

## Data Availability

The datasets used and/or analyzed during the current study are available from the corresponding author upon reasonable request.
